# Gut Microbiota of Freshwater Gastropod (*Bellamya aeruginosa*) Assist the Adaptation of Host to Toxic Cyanobacterial Stress

**DOI:** 10.3390/toxins15040252

**Published:** 2023-03-29

**Authors:** Hongfang Liu, Xianming Yang, Wen Yang, Zhongming Zheng, Jinyong Zhu

**Affiliations:** Laboratory of Aquatic Ecology, School of Marine Science, Ningbo University, No. 169, South Qixin Road, Ningbo 315832, China

**Keywords:** *Bellamya aeruginosa*, *Microcystis aeruginosa*, microcystins, gut microbiota, co-occurrence networks

## Abstract

Gut microbes play a critical role in helping hosts adapt to external environmental changes and are becoming an important phenotype for evaluating the response of aquatic animals to environmental stresses. However, few studies have reported the role that gut microbes play after the exposure of gastropods to bloom-forming cyanobacteria and toxins. In this study, we investigated the response pattern and potential role of intestinal flora in freshwater gastropod *Bellamya aeruginosa* when exposed to toxic and non-toxic strains of *Microcystis aeruginosa*, respectively. Results showed that the composition of the intestinal flora of the toxin-producing cyanobacteria group (T group) changed significantly over time. The concentration of microcystins (MCs) in hepatopancreas tissue decreased from 2.41 ± 0.12 on day 7 to 1.43 ± 0.10 μg·g^−1^ dry weight on day 14 in the T group. The abundance of cellulase-producing bacteria (*Acinetobacter*) was significantly higher in the non-toxic cyanobacteria group (NT group) than that in the T group on day 14, whereas the relative abundance of MC-degrading bacteria (*Pseudomonas* and *Ralstonia*) was significantly higher in the T group than that in the NT group on day 14. In addition, the co-occurrence networks in the T group were more complex than that in the NT group at day 7 and day 14. Some genera identified as key nodes, such as *Acinetobacter*, *Pseudomonas,* and *Ralstonia*, showed different patterns of variation in the co-occurrence network. Network nodes clustered to *Acinetobacter* increased in the NT group from day 7 to day 14, whereas the interactions between *Pseudomonas* and *Ralstonia* and other bacteria almost changed from positive correlations in the D7T group to negative correlations in the D14T group. These results suggested that these bacteria not only have the ability to improve host resistance to toxic cyanobacterial stress by themselves, but they can also further assist host adaptation to environmental stress by regulating the interaction patterns within the community. This study provides useful information for understanding the role of freshwater gastropod gut flora in response to toxic cyanobacteria and reveals the underlying tolerance mechanisms of *B. aeruginosa* to toxic cyanobacteria.

## 1. Introduction

Over the past decades, cyanobacterial harmful algal blooms (CHABs) have become a significant environmental issue on a global scale in the context of accelerating climate change. Bloom-forming cyanobacteria can produce a number of toxic secondary metabolites, among which the potent hepatotoxic microcystins (MCs) are the most common in the environment [1]. MCs can be greatly enriched in tissues via ingestion in the digestive tract and absorption on the body surface of aquatic animals [2] and consequently cause a series of adverse effects on the organism [3]. The high spatiotemporal overlap between harmful cyanobacterial bloom outbreaks and the survival and reproductive activities of freshwater gastropods [4] results in the toxic cyanobacteria having a direct impact on snails. Although numerous studies have reported a number of adverse effects on snails caused by bloom-forming cyanobacteria [3], mollusks can be enriched in higher levels of MCs compared with mammals and fish [5], and MCs do not appear to have a significant effect on gastropod reproductive capacity [6]. By contrast, bivalve mollusks, which are also vigorous filter feeders, are unable to survive from cyanobacterial blooms as well as snails [7]. These findings indicate that freshwater gastropods have stronger adaptability and tolerance to toxic cyanobacteria than their counterparts. The underlying mechanisms of snail tolerance to bloom-forming cyanobacteria have been studied from ecological [8], physiological–biochemical [9,10], and omics [11,12] perspectives. However, all these studies have explored the possible adaptive mechanisms from the gastropod itself, without considering the role and function played by the symbiotic microorganisms when exposed to cyanobacterial blooms from the perspective of the holobiont.

As an important group of symbiotic bacteria, many studies have found that intestinal microbiota can play an important role in the life activities of aquatic animals, including freshwater gastropods [13]. In recent years, an increasing number of studies have focused on the composition of the gut microbiota of freshwater snails [14,15] and revealed the heterogeneity of snail gut microbiota across gut sections [16], gender and developmental stages [17], host habitats [18,19], host species [20,21], and dietary conditions [22]. Unfortunately, however, these studies have not explored the possible role of gut flora in the host’s response to environmental changes, but studies have confirmed that gut flora can influence the ability of aquatic animals to tolerate toxic cyanobacterial [23]. Thus, snail gut flora may also have a similar role in the host response to toxic cyanobacterial stress.

*Bellamya aeruginosa* is a native freshwater snail in China, mainly distributed in the littoral zone of various freshwater bodies, which plays an important role in aquatic ecosystems while also being considered an ideal sentinel species for environmental monitoring and assessment [24]. The dynamic characteristics and response patterns of the *B. aeruginosa* gut microbiota to toxic cyanobacterial blooms were investigated in our previous field study. Given the complexity and variability of the field environment, gut microbes are affected by variables that are unobservable or difficult to quantify, and the association of gut microbiota with bloom-forming cyanobacteria and their toxins is difficult to identify [19]. Therefore, we directly examined the effect of MC-producing cyanobacteria and non-MC—producing cyanobacteria on snail intestinal flora under controlled experimental conditions in simulated bloom waters to test the following hypotheses: (1) the composition, community assembly processes, and co-occurrence network of the gut microbes between the NT and T groups significantly differ, and the main factor contributing to these differences will be MCs; (2) these differences will likely be present to help the snails digest algae and degrade MCs, as well as to adapt to the toxic cyanobacterial stress.

## 2. Results

### 2.1. MC Concentration in Hepatopancreas Tissue

We determined the concentration of MCs in hepatopancreatic tissue of *B. aeruginosa* in the two groups using an ELISA kit on days 0, 7, and 14, respectively. MCs were only detected in the T group on days 7 and 14. To further test for a significant difference in the algal toxin concentrations in the T group at these two time points, an unpaired two-tailed Student’s *t*-test was performed. The results showed that the concentration of MCs decreased sharply from 2.41 ± 0.12 on day 7 to 1.43 ± 0.10 μg·g^−^^1^ dry weight (DW) on day 14 (*t*-test, *p* < 0.0001; Table 1).

### 2.2. Changes in Intestinal Microbiota Composition

**Figure 1:**
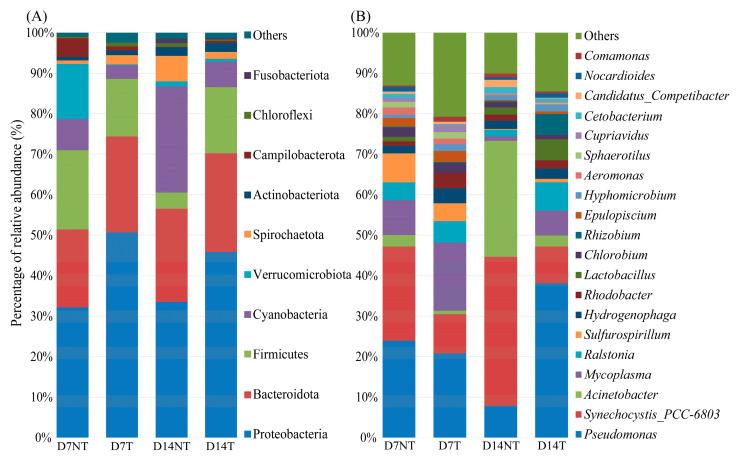
Relative abundance of each group of gut microbes at the phylum (**A**) and genus (**B**) levels. Abbreviations: D7NT, day 7 of the non-toxic cyanobacteria group; D7T, day 7 of the toxin-producing cyanobacteria group.

A total of 6,220,221 sequencing reads were acquired after quality control (Table S1) and clustered by USEARCH software, resulting in 6,881 ZOTUs (Table S2). These identified ZOTUs were annotated into 51 bacterial phyla, 330 families, and 616 genera (Table S2). At the phylum level, the core flora of *B. aeruginosa* were Proteobacteria, Firmicutes, and Bacteroidota in both the D7NT and D7T groups, with 32.16%, 19.55%, and 19.26% in the D7NT group and 50.65%, 14.23%, and 23.71% in the D7T group, respectively. The gut flora of the D14NT group were dominated by Proteobacteria (33.49%), Cyanobacteria (26.19%), and Bacteroidota (23.05%), and the core flora were Proteobacteria (45.85%), Bacteroidota (24.38%), and Firmicutes (16.30%) in the D14T group (Figure 1A and Table S3). At the genus level, the gut flora of the D7NT group were dominated by *Pseudomonas* (23.60%) and *Synechocystis_PCC-6803* (23.27%), and the core flora were *Pseudomonas* (20.77%) and *Mycoplasma* (16.79%) in the D7T group. The most abundant genera were *Synechocystis_PCC-6803* (36.79%) and *Acinetobacter* (28.98%) in the D14NT group, whereas the core flora were *Pseudomonas* (38.13%) and *Synechocystis_PCC-6803* (9.08%) in the D14T group (Figure 1B and Table S4).

### 2.3. Diversity Analysis of Intestinal Microbiota

**Figure 2:**
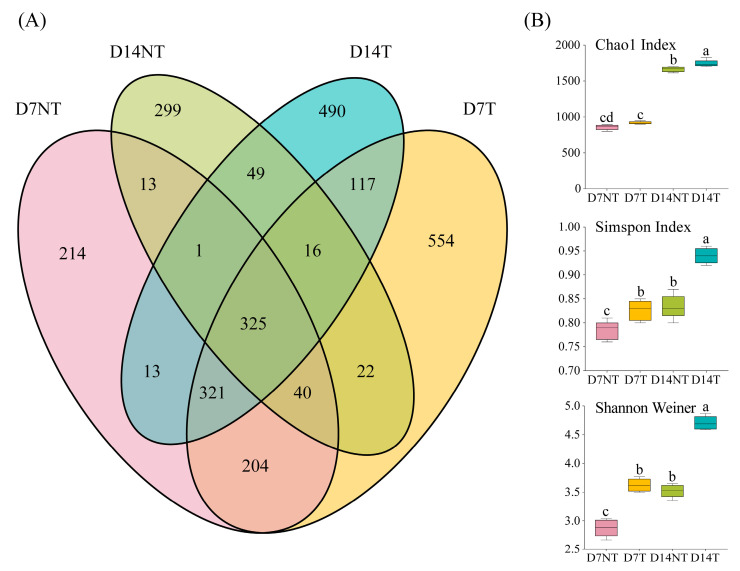
Distribution of the number of ZOTUs and comparison of alpha diversity indices between the two groups. (**A**) Venn diagram shows the common and unique ZOTUs between the NT and T groups at two time points. (**B**) Comparison of alpha diversity indices of gut microbial communities between the two groups. Different lowercase letters indicate significant differences between groups (*p* < 0.05).

The results of a Venn analysis showed that 890 (48.37%) ZOTUs were common to the two groups on day 7, and 241 (13.10%) and 709 (38.53%) ZOTUs belonged uniquely to the D7NT and D7T groups, respectively. However, only 391 (22.92%) ZOTUs were shared by the two groups on day 14, and 374 (21.92%) and 941 (55.16%) ZOTUs were uniquely classified into the D14NT and D14T groups, respectively (Figure 2A). The results showed that the Chao1 index of the D14T group was significantly higher than that in the D14NT group (ANOVA, *p* < 0.05; Figure 2B), and the diversity indices (Simpson and Shannon) were significantly higher in the T group than those in the NT group at two time points (ANOVA, *p* < 0.05; Figure 2B). 

**Figure 3:**
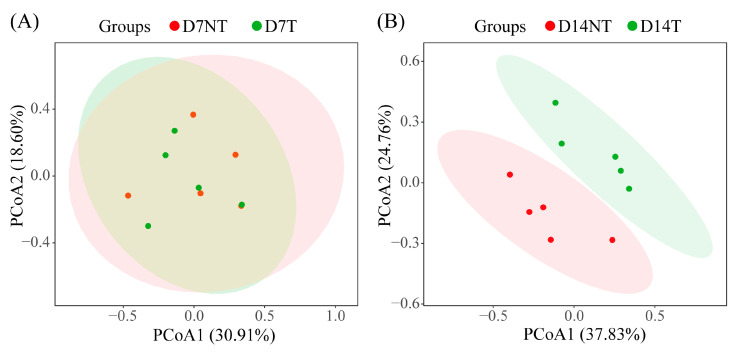
PCoA plots of the gut microbiota in both groups on day 7 (**A**) and day 14 (**B**). Ellipses indicate 95% confidence interval, and the percentages in parentheses are the proportion of variation explained by the PCoA axis.

To test for a statistically significant difference in the community composition and structure of the gut microbiota between the two groups at the same time point, PCoA and statistical tests were performed. The results showed no significant difference in the composition and structure of the intestinal flora between the two groups on day 7, and the sum of the interpretation of the two axes was 49.51% (PERMANOVA, ANOSIM, and MRPP tests, *p* > 0.05; Figure 3A and Table 2). However, a significant difference was noted in the composition and structure of the intestinal flora between the two groups on day 14, with the sum of the percentages of explanation for the two axes being as high as 62.59% (PERMANOVA, ANOSIM, and MRPP tests, *p* < 0.05; Figure 3B and Table 2).

### 2.4. Biomarker Analysis

**Figure 4:**
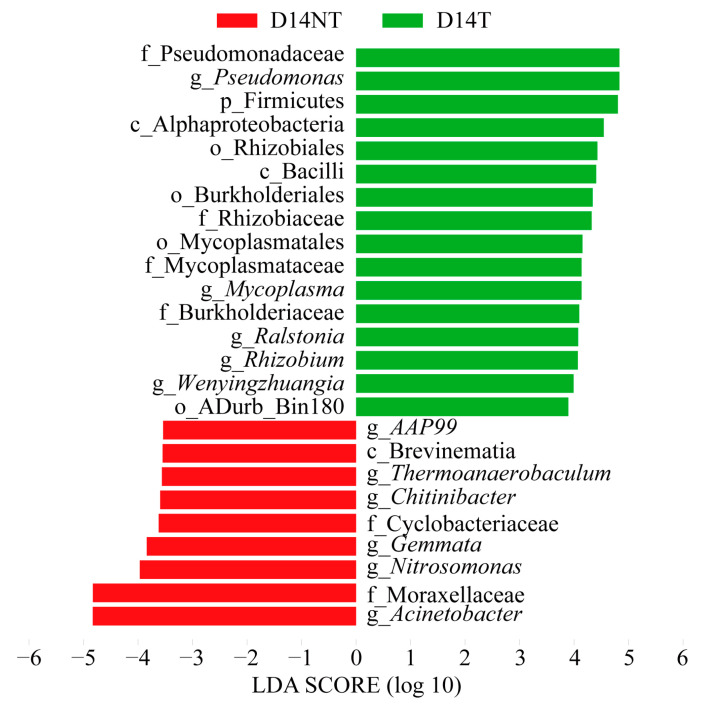
Differential taxa of gut microbes in the two groups on day 14 (LDA > 3.5, *p* < 0.05). Note that the genus *Allorhizobium-Neorhizobium-Pararhizobium-Rhizobium* was replaced by the abbreviation *Rhizobium* for overall aesthetics.

**Figure 5:**
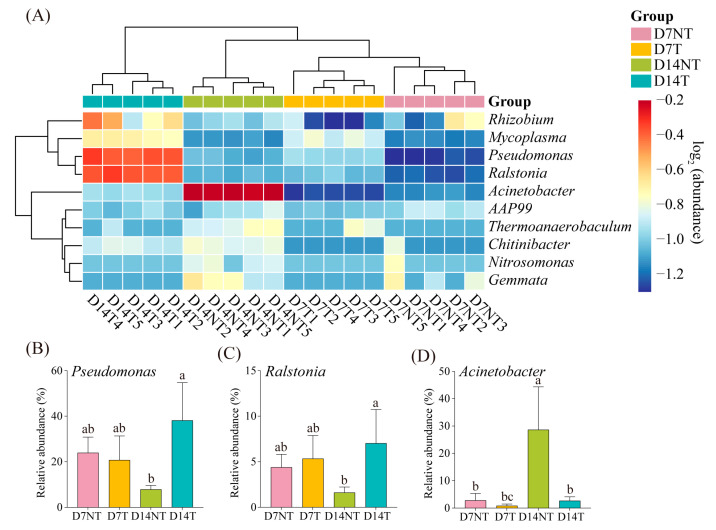
(**A**) Heat map showing the abundance of biomarkers at the genus level in different groups. The abundance data were log_2_ transformed. Both samples and abundance were clustered based on Euclidian distances. A one-way ANOVA with Holm–Sidak’s multiple comparisons test was performed to test for a significant difference in the proportion of *Pseudomonas* (**B**), *Ralstonia* (**C**), and *Acinetobacter* (**D**) between the groups at two time points. Abbreviations: D7NT1, the first sample of the non-toxic cyanobacteria group on day 7; D7T1, the first sample of the toxin-producing cyanobacteria group on day 7. Different lowercase letters indicate significant differences between groups (*p* < 0.05).

To further identify statistically significant biomarkers in the NT and T groups, LEfSe analysis based on the bacterial taxon was performed. Our results showed no enriched bacteria in the two groups on day 7. However, 25 biomarkers (LDA score > 3.5) were screened in the two groups on day 14 (Figure 4). Specifically, nine taxa were abundant in the D14NT group, including the family Moraxellaceae and the genera *Acinetobacter*, *Nitrosomonas*, and *Gemmata*, whereas 16 taxa were enriched in the D14T group, including the phylum Firmicutes, the class Bacilli, the family Pseudomonadaceae, and the genera *Pseudomonas* and *Ralstonia*. The heat map showed that *Pseudomonas* and *Ralstonia* were most abundantly represented in the D14T group, whereas *Acinetobacter* was most abundant in the D14NT group (Figure 5A). To further test whether there was a significant difference in the proportion of the relative abundances of these three biomarkers between groups, a one-way ANOVA with Holm–Sidak’s multiple comparisons test was performed. The results showed that there was no significant difference in the relative abundance of *Pseudomonas*, *Ralstonia*, and *Acinetobacter* between the D7NT and D7T groups (ANOVA, *p* > 0.05; Figure 5B–D). However, there was a significantly higher percentage of *Pseudomonas* and *Ralstonia* in the D14T group compared with the D14NT group (ANOVA, *p* < 0.05; Figure 5B,C). The relative abundance of *Acinetobacter* in the D14NT group was significantly higher than that in the D14T group (ANOVA, *p* < 0.05; Figure 5D). 

### 2.5. Gut Microbiota Assembly Process Analysis

**Figure 6:**
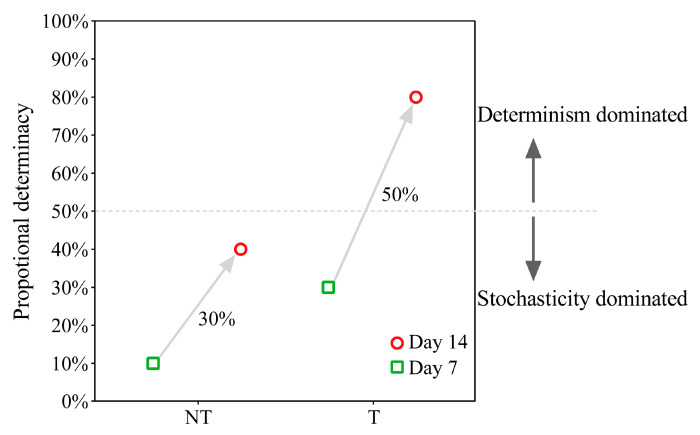
Changes in the proportion of deterministic and stochastic processes in the community assembly of the two groups of intestinal microorganisms. The gray dashed line in the figure indicates the equal role of both. Above or below the line represents the dominance of deterministic or stochastic processes, respectively. The percentage next to the gray arrow in the graph indicates the increase in the deterministic process.

MNTD analyses was applied to quantify the determinants of intestinal bacterial community assembly processes in the two groups. The results showed that the community assembly process was dominated by stochastic processes in the D7NT and D7T groups, with deterministic processes contributing 10% and 30% (Figure 6 and Table S5, day 7), respectively. The community assembly process was still dominated by stochastic processes in the D14NT group, with deterministic processes contributing 40% (Figure 6 and Table S5, D14NT group), whereas the community assembly process in the D14T group was dominated by deterministic processes, with deterministic processes contributing 80% (Figure 6 and Table S5, D14T group).

### 2.6. Co-Occurrence Patterns of Intestinal Flora

**Figure 7:**
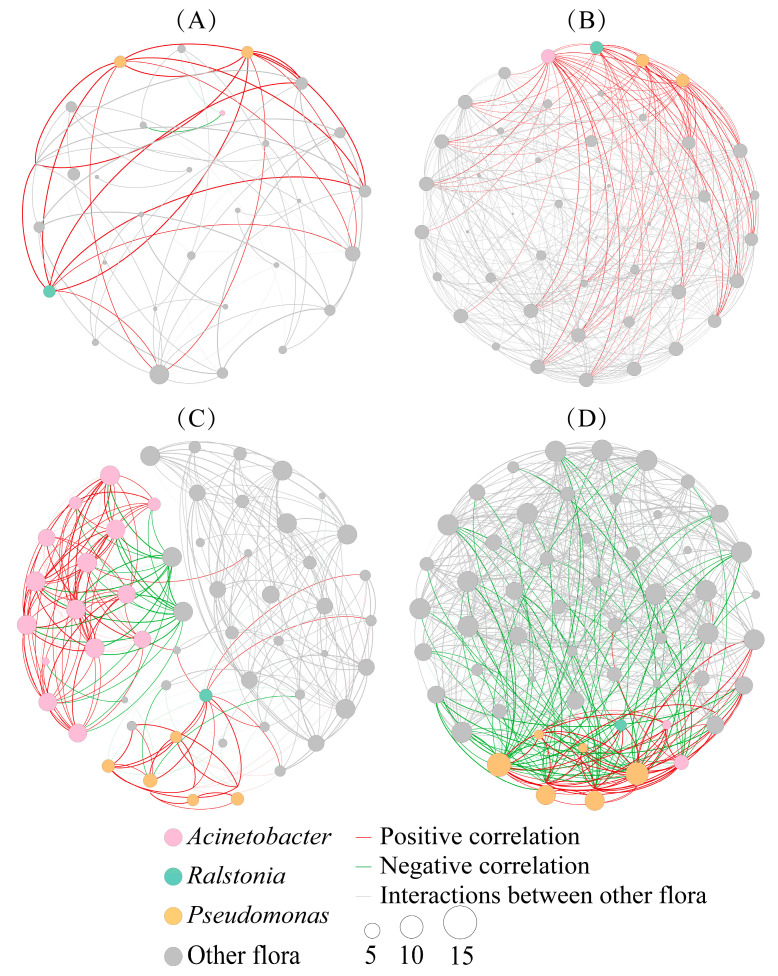
Co-occurrence network map for ZOTUs at the genus level in both groups. (**A**) D7NT group; (**B**) D7T group; (**C**) D14NT group; (**D**) D14T group. A connection stands for a strong (Spearman’s |*r*| ≥ 0.7) and significant (*p* < 0.05) correlation. The genera *Pseudomonas*, *Ralstonia,* and *Acinetobacter* are colored in orange, green, and pink, respectively, and the remaining nodes are colored in grey. The size of the nodes represents the degree, and the red and green lines represent positive and negative correlations, respectively. The thickness of the line represents the strength of correlation between the nodes.

To clarify the interactions between bacteria, ZOTUs were selected for the co-occurrence network analysis at the genus level. Some important topological parameters in the two groups were also counted. The results showed that the co-occurrence networks were more complex in the T group than that in the NT group at two time points (Figure 7). This was also reflected on some key topological parameters. Compared to the D7NT group, the D7T group had higher values on the Nodes (47 and 29 nodes in the D7T and D7NT group, respectively), Edges (383 and 65 edges), Average degree (16.298 and 4.483), Average clustering coefficient (ACC, 0.800 and 0.665), and Density (0.354 and 0.160), and lower values on the Modularity (0.305 and 0.535 in the D7T and D7NT group, respectively), Diameter (6 and 8), and Average path length (APL, 2.156 and 3.345), indicating that the co-occurrence network of the D7T group was more complex than that of the D7NT group (Table 3, D7NT, and D7T groups). Similar results were also observed between the D14NT and D14T groups. The co-occurrence networks in the D14NT and D14T groups consisted of 55 nodes with 280 edges and 65 nodes with 579 edges, respectively. The Average degrees and ACC were 10.182 and 0.759 in the D14NT group compared to 21.055 and 0.766 in the D14T group, and the Density was 0.189 in the D14NT group and 3.390 in the D14T group. The Modularity and Diameter were 0.621 and 8 in the D14NT group compared to 0.247 and 4 in the D14T group, and the APL was 3.452 in the D14NT group and 1.834 in the D14T group (Table 3, D14NT and D14T groups). Our results also showed a complex co-occurrence network structure for each group over time, especially in the T group (Figure 7 and Table 3, D7T and D14T groups). Three bacteria in the two groups showed distinct different patterns of variation in the co-occurrence network diagram on the time scale. The number of nodes clustered to *Acinetobacter* increased in the D14NT group compared to the D7NT group (Figure 7A,C), whereas the interactions between *Pseudomonas* and *Ralstonia* and other bacteria almost changed from positive correlations in the D7T group to negative correlations in the D14T group (Figure 7B,D). 

**Figure 8:**
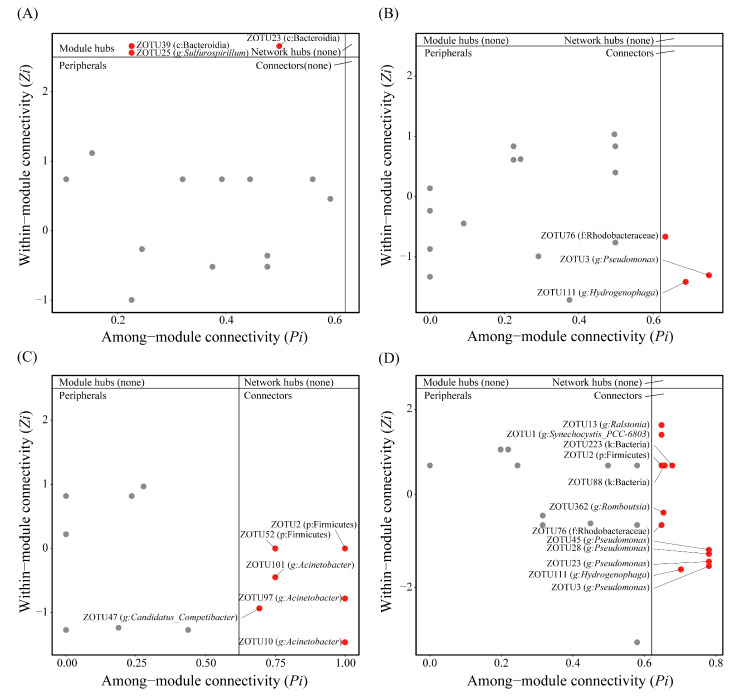
*Zi*-*Pi* plots showing the distribution of ZOTUs with their topological roles in each group. (**A**) D7NT group; (**B**) D7T group; (**C**) D14NT group; (**D**) D14T group. The threshold values of *Zi* and *Pi* for classifying nodes are 2.5 and 0.62, respectively.

To further evaluate the topological role of identified nodes in the network, the within-module connectivity (Zi) and among-module connectivity (Pi) values were used to separate network nodes into four ecological modules, including module hubs, network hubs, peripherals, and connectors. We performed Zi-Pi analysis for the D7NT, D7T, D14NT, and D14T groups, respectively. Our results showed that there were three network nodes that were assigned to module hubs in the D7NT group (Figure 8A), and three network nodes were assigned to connectors in the D7T group, including one connector clustered to *Pseudomonas* (Figure 8B). Six network nodes were identified as connectors in the D14NT group, including three connectors clustered to *Acinetobacter* (Figure 8C), and twelve network nodes were identified as connectors in the D14T group, including four connectors clustered to *Pseudomonas* and one connector clustered to *Ralstonia* (Figure 8D). 

## 3. Discussion

### 3.1. Toxic-Producing Cyanobacteria Alter the Intestinal Flora of B. aeruginosa 

Proteobacteria and Bacteroidota were the core flora in all groups of intestinal flora of *B. aeruginosa* at the phylum level (Figure 1A), which was similar to previous results for both freshwater [15,16,21] or seawater snails [25]. There was no significant difference in the relative abundance of some genera between the two groups on day 7, such as *Pseudomonas*, *Ralstonia,* and *Acinetobacter* (Figure 5B–D, day 7). However, the relative abundance of *Pseudomonas* and *Ralstonia* in the D14T group was significantly higher than that in the D14NT group, whereas the relative abundance of *Acinetobacter* was significantly lower than that in the D14NT group (Figure 5B–D, day 14). In addition, the D14NT group had a higher relative abundance of *Acinetobacter* compared to the D7NT group (Figure 5D, D7NT and D14NT groups). These results seem to indicate that different algae have different effects on the gut microbes of snails, and the effects increase over time. PCoA also showed there was no significant difference in the composition of the gut microbiota between the NT and T groups on day 7 (Figure 3A), but the microbiota was different between the two groups on day 14 (Figure 3B). Even though the timing of microbial community differences between groups (Figure 3) and the peak of MC accumulation (Table 1) were not synchronized due to possible lags in intestinal flora changes, the difference in the composition and structure of intestinal flora in *B. aeruginosa* could be largely attributed to MCs. This is because the different strains of *M. aeruginosa* do not differ significantly in morphology, cell structure, and nutritional value but only in their ability to produce toxins [26]. Moreover, MC enrichment was observed in the hepatopancreas of snails in the T group at day 7 and day 14, whereas no accumulation was observed in the NT group at day 7 and day 14 (Table 1).

In our field study, we also observed a significant effect of toxic cyanobacterial blooms on the gut flora of *B. aeruginosa*, which was accompanied with a decrease in community diversity [19]. These findings were consistent with the fact that a reduction in gut microbial diversity also occurs in aquatic animals and even snails after toxicant exposure [27,28,29]. However, feeding toxic *M. aeruginosa* instead increased the richness and diversity of gut microbes in the present study (Figure 2B, D7T and D14T groups), which was similar to the result of a previous study that gut microbial richness and diversity increased in mice after MC exposure [30]. Since the experimental animals and toxicant in our field study were the same as that in this study, this conflicting result cannot be explained by the differences in the type of toxicant and experimental animals. Possible reasons may be due to the different experimental conditions; compared with the controlled indoor experiments, there are more complex influencing factors in the field, including unobserved or difficult-to-observe environmental factors and complex and variable optional food sources that can directly affect the flora [8,19]. Furthermore, our results showed that the D14NT group was significantly different from the D7NT group in both richness and diversity indices (Figure 2B, D7NT and D14NT groups). The reason for this result is most likely due to the fact that *M. aeruginosa*, as an undesirable food [31], influenced the composition and structure of the gut microorganisms in the NT group. This effect may become progressively greater over time and thus eventually lead to significant differences in the richness and diversity of the gut flora in the NT group at the two time points. Feeding the freshwater pulmonate snail *Planorbella trivolvis* with high and low cellulose foods, respectively, also resulted in significant differences in diversity indices between the two groups of gut microorganisms [22].

### 3.2. Gut Microbiota Assist B. aeruginosa to Adapt to Toxic Cyanobacterial Stress

As mentioned before, *M. aeruginosa* is not considered an ideal food source for aquatic animals [31], but the freshwater snail *B. aeruginosa* is only able to ingest and digest *M. aeruginosa* to some extent [8,32]. One important reason for the poor digestibility of *M. aeruginosa* is that the impermeable cell walls of the algae prevent the release of cellular contents [31]. The breakdown of the algal cell wall in the aquatic animals’ digestive tract can only be achieved by three pathways: mechanical trituration, acid hydrolysis, and enzymatic digestion [33]. Unfortunately, *B. aeruginosa* not only does not possess grinding organs (such as crop) and an acidic gastrointestinal environment [34], but it also does not have the capacity for the endogenous synthesis of relevant enzymes [35]. The gut microbiota make the last route possible. Several studies have found that the intestinal microbes of gastropods can help hosts enhance algal food digestion by degrading alginate [36] and cellulose [22]. Our study also found that there was a higher relative abundance of *Acinetobacter* in the NT group than that in the T group on day 14 (Figure 5D, day 14). Moreover, the relative abundance of *Acinetobacter* in the NT group increased sharply from 2.80% on day 7 to 28.98% on day 14 (Figure 5D and Table S4, D7NT and D14NT groups). The main reason for these results was that *Acinetobacter*, as the cellulase-producing bacterium [37], may help the snail to digest the cell wall of *M. aeruginosa* to some extent when *B. aeruginosa* responded to the undesirable algal food. However, there was a higher abundance of *Pseudomonas* and *Ralstonia* in the T group than that in the NT group on day 14 (Figure 5B,C, day 14). The presence of MCs may be responsible for this contrasting outcome of intestinal microbiota succession between the two groups. Some *Pseudomonas* species and *Ralstonia* species have been reported to degrade MCs [38,39]. MCs, as an intracellular toxin, are released only after cell death or damage accompanied with cell structure disruption [40]. This makes it a dilemma for the holobiont formed by aquatic animals and their gut microbiota whether to utilize the organic matter inside the algal cells in the face of toxic-producing cyanobacteria. If the intestinal flora help the host achieve cell wall degradation, it would mean toxin release; not doing so might result in a lack of sufficient energy intake for the host. This is probably one of the reasons why toxic cyanobacteria, such as *M. aeruginosa*, evolved toxins as an anti-predator mechanism [41]. However, the relatively higher abundance of MC-degrading bacteria in the D14T group compared with the D14NT group (Figure 5B,C, day 14) was accompanied with an eventual decline in MCs’ accumulation (Table 1), suggesting that intestinal microbiota appeared to provide a way to subtly solve this conundrum by maintaining MC-degrading bacteria in the T group and increasing cellulase-producing bacteria in the NT group (Figure 4).

The co-occurrence network of intestinal microbiota in the T group was more complex than that in the NT group both at day 7 and day 14. (Figure 7). In addition, the co-occurrence network of each group of snail gut microbes seemed to become more complex over time, especially in the T group (Figure 7, D7T and D14T groups). These results were also reflected by some key topological parameters (Table 3). These results may be due to the fact that both the undesirable algal food and toxic cyanobacterial stress appeared to help the intestinal flora to maintain the stability of their own community structure, but the effects of toxic cyanobacterial stress seemed to be stronger than those of the undesirable algal food. Depending on the micro-environment, some key floras can choose different strategies to shape the flora in favor of their own growth, thereby significantly affecting the composition and function of the community in the host [42]. This seems to be evident from the dynamics of gut microbiota complexity and relationships. In our study, *Acinetobacter* was identified as a key node in the D14NT group (Figure 8C), and *Pseudomonas* and *Ralstonia* were identified as key nodes in the D14T group (Figure 8D). Our results also showed that these key nodes in different groups showed different patterns of variation in the co-occurrence network. The network nodes clustered to *Acinetobacter* increased in the D14NT group compared with the D7NT group (Figure 7A,C). However, not only did more *Pseudomonas* nodes appear, and more complex interactions were found among *Pseudomonas* and *Ralstonia* and other bacteriain the D14T group compared with the D7T group, but also many interactions changed from positive to negative (Figure 7B,D). The main factor affecting the co-occurrence network of the snail gut flora in the NT group was the undesirable algal food, whereas it was toxic cyanobacterial stress in the T group. Many studies have found that host exposure to environmental stresses, such as toxic exposure and dietary alterations, does lead to more nodes or more complex interactions of gut flora and even to shifts in the positive and negative relationships between some key bacteria and other bacteria [43,44,45]. *Pseudomonas* and *Ralstonia*, as MC-degrading bacteria [38,39], may not only have the ability to increase host tolerance to MCs by themselves, but the co-occurrence network revealed that these bacteria may also influence host resistance to toxic cyanobacterial stress by affecting the entire bacterial community.

The increase in negative interactions is often considered a way to improve the stability of disturbed bacterial communities [46]. Therefore, it is likely that these changes in the interactions between *Pseudomonas* and *Ralstonia* and other bacteria in the T group from day 7 to day 14 (Figure 7B,D) are intended to improve the stability of the overall gut community structure as a means of helping the host to better adapt to the toxic algal environment. Topological features of a network, such as increases in edges and nodes, exactly reflect the increased stability in the T group (Table 3). Alterations in interactions among bacteria also affect the community assembly mechanism of the gut microbiome [47], which is also evidenced by our results that the community assembly process in the T group changed from being dominated by stochastic processes on day 7 to being dominated by deterministic processes on day 14 (Figure 6 and Table S5, D7T and D14T groups). However, the community assembly process was all dominated by stochastic processes in the NT group at two time points (Figure 6 and Table S5, D7NT and D14NT groups). One of the reasons for this result may be that the toxic cyanobacterial stress had a stronger effect than the undesirable algal food on the gut microbes of *B. aeruginosa*. These above results indicated that these key bacteria influence the snail gut microbiota by regulating their association with other bacteria or increasing nodes, allowing the flora to play an important role in helping the host deal with different environmental stresses, such as the undesirable algal food or toxic cyanobacterial stress.

## 4. Conclusions

Our study confirmed that the intestinal microbiota of gastropods can respond to bloom-forming cyanobacteria exposure in various aspects, including composition, diversity, co-occurrence network and community assembly mechanisms. MCs are likely to be the main factors contributing to these dynamics in flora. *Pseudomonas* and *Ralstonia*, as MC-degrading bacteria, play an important role in enhancing snail host adaptation to toxic-producing cyanobacterial stress in the T group. They may not only have the ability to promote the degradation of MCs but may also be able to assist host adaptation to toxic cyanobacteria by modulating interaction patterns within the community. Moreover, *Acinetobacter*, as a cellulase-producing bacteria, may help the snail host to digest *M. aeruginosa* in the NT group. These results mainly reveal the role of gut microbiota in the response of freshwater gastropods to toxic cyanobacterial stress and provide important information to explain the tolerance mechanisms of *B. aeruginosa* to toxic *M. aeruginosa*.

## 5. Materials and Methods

### 5.1. Biological Materials

The freshwater snail, *B. aeruginosa*, was collected from the Yinzhou Park in Ningbo City (E 121°55′, N 29°81′), Zhejiang Province, China. All snails with uniform size (2.0 ± 0.1 cm of shell length) were selected and acclimated in aquariums (34 cm × 14 cm × 22 cm) for 7 days before the experiment. During this period, all snails were fed with *Chlorella vulgaris,* and the water was refreshed once a day.

All algal species, including the non-toxic-producing strain *M. aeruginosa* FACHB-469 without the MC synthetase gene [48], the toxic-producing strain *Microcystis aeruginosa* FACHB-905, producing MC-LR about 0.61 μg per 10^7^ viable cells [49], and *C. vulgaris* NMBlud2006-2, were provided by the Laboratory of Aquatic Ecology, Ningbo University. *M. aeruginosa* was cultured in 1-L Erlenmeyer flasks axenically using BG-11 medium (product composition information is shown in Table S6) at a constant temperature (25 ± 1 °C) and photoperiod (12 h light: 12 h dark cycle) and irradiance of 36 µE·m^−2^·s^−1^. *C. vulgaris* was mass cultured in a photobioreactor using NMB3# medium (product composition information is shown in Table S7) at 25 °C under natural light with aeration.

### 5.2. Experimental Design

All experiments were performed at a constant room temperature and natural irradiance. After the end of adaptive feeding, 30 snails were equally and randomly distributed into the two groups: the non-toxic cyanobacteria group (exposed to non-toxic *M. aeruginosa* FACHB-469, represented as NT group, cell density: 1.8 × 10^7^ cells·mL^−1^) and the toxin-producing cyanobacteria group (exposed to toxin-producing *M. aeruginosa* FACHB-905, represented as T group, cell density: 1.8 × 10^7^ cells·mL^−1^). The snails were fed indoors for 14 days according to the established algal concentrations of each group. The water was refreshed once a day, and the algal concentrations were adjusted every day. As the snail numbers decreased, the algal suspension was reduced accordingly, and 300 mL of algal suspension per snail was maintained throughout the experiment. Five living snails were randomly removed from each group at days 0, 7, and 14 after feeding, respectively. Then, their intestinal and hepatopancreas tissues were dissected and removed and stored at −80 °C until DNA extraction and microcystins (MCs)’ concentration determination using an ELISA kit, respectively.

### 5.3. Determination of MC Concentration

The MCs were extracted according to a previous study [50]. The concentrations of MCs in the hepatopancreas were determined using an ELISA Microcystin Panel Kit (Beacon Analytical Systems, Portland, ME, USA; detection limit 0.1 μg/L) according to the manufacturer’s instructions. Although the kit could detect many species of MCs, it could not distinguish between MC-LR and other variants. Thus, the concentrations of MCs in this study were considered to be equivalents of MC-LR. Moreover, we determined matrix effects and recoveries for MC extraction, and the results we obtained were in line with previous results [19]. The average extraction efficiency was 91.6%, and the matrix effect was almost negligible due to the small difference in results between methanol and the matrix, with an average of 4.3%. Due to the high recovery efficiency and the low matrix effect, the concentrations of MCs in this study were expressed as actual measurements, and no other additional calculations were performed.

### 5.4. DNA Extraction, Amplification and Sequencing

The method of DNA extraction was performed according to a previous study [19]. The variable regions V3-V4 of bacteria were amplified by PCR using the universal primer 338F (5′-ACTCCTACGGGAGGCAGCAG-3′) and 806R (5′-GGACTACHVGGGTWTCTAAT-3′) [51]. Primers were obtained from Invitrogen (Invitrogen, Carlsbad, CA, USA). The PCR reaction consisted of the following procedure: initialization at 94 °C for 5 min; 30 cycles, denaturation at 94 °C for 30 s, annealing at 52 °C for 30 s, extension at 72 °C for 30 s; and then final extension at 72 °C for 10 min. Sequencing libraries were constructed according to the standard procedure of NEBNext^®^ Ultra™ II DNA Library Prep Kit for Illumina^®^ (New England Biolabs, Ipswich, MA, USA). Finally, the libraries were sequenced using the Illumina Nova 6000 platform (Guangdong Magigene Biotechnology Co., Ltd., Guangzhou, China).

### 5.5. Processing of Sequencing Data

Sequenced paired-end reads were processed using USEARCH (Version 11) [52,53]. The unique sequence reads were obtained with the fastx_uniques function, and then noise reduction was performed with the UNOISE3 algorithm at unoise_alpha = 4 and minsize = 6. This produced a list of zero-radius operational taxonomic units (ZOTUs). A ZOTU abundance table was generated by mapping the total number of reads to the ZOTU list using the otutab function with an identity threshold of 0.97. A representative sequence of each ZOTU was assigned to the taxonomic group using the RDP classifier in the SILVA database (version 138) with a similarity of 99%.

### 5.6. Bioinformatics and Statistical Analyses 

The data were pre-processed in Microsoft Excel 2016, and all analyses were performed in R software (version 4.1.2) and visualized using the package “ggplot2” [54] unless specifically stated. An unpaired two-tailed Student’s *t*-test was applied in GraphPad software (version 9.4.0) to test for statistically significant differences in MC concentrations between day 7 and day 14 for the T group. The stacking diagram was created with Microsoft Excel 2016. The Venn diagram was drawn in R with the package “VennDiagram” [55]. Alpha diversity indices (Chao1, Shannon, and Simpson indices) were calculated in R using the package “vegan” [56]. A one-way analysis of variance (ANOVA) with Tukey’s multiple comparisons test was used to test for a significant difference in alpha diversity indices between groups by using GraphPad. Principal coordinate analysis (PCoA)-based weighted unifrac distance was conducted in R with the package “phyloseq” [57]. Permutational multivariate analysis of variance (PERMANOVA), analyses of similarity (ANOSIM), and multi-response permutation procedure (MRPP) were performed to test whether there was a statistically significant difference in the community composition and structure between groups with the adonis, anosim, and mrpp functions from the package “vegan”. Linear discriminant analysis (LDA) effect size (LEfSe) analysis [58] was carried out on the website (https://huttenhower.sph.harvard.edu/galaxy/, accessed on 21 December 2021). The heatmap was created in R with the package “pheatmap” [59], and a one-way ANOVA with Holm–Sidak’s multiple comparisons test was used to test whether there was a significant difference in the abundance of biomarkers between the two groups in GraphPad. To compare the effects of toxic-producing cyanobacteria on the assembly of the gut microbiota, the mean nearest taxon distance (MNTD) analysis was applied in R using the packages “picante” and “ape” [60,61]. Co-occurrence network analysis was performed in R with the packages “Hmisc” [62] and “igraph” [63] and visualized in Gephi software (version 0.9.2) using the Fruchterman–Reingold layout. ZOTUs with a total relative abundance of more than 0.01 in all samples were selected for co-occurrence network analysis. Spearman’s correlation was calculated to quantify correlations, with a correlation coefficient r ≥ 0.7 and *p *< 0.05. Within-module connectivity (*Zi*) and among-module connectivity (*Pi*) thresholds were used to classify the ecological roles of individual nodes in the network [64]. Theoretically, network nodes were classified into four ecological modules: peripherals (*Zi*  ≤  2.5 and *Pi*  ≤  0.62), connectors (*Zi*  ≤  2.5 and *Pi*  >  0.62), module hubs (*Zi*  >  2.5 and *Pi*  ≤  0.62), and network hubs (*Zi*  >  2.5 and *Pi*  >  0.62) [65]. In general, the nodes that appear in connectors, module hubs, and network hubs are considered keystone taxa in the network [66].

## Figures and Tables

**Table 1:** Concentration of MCs in hepatopancreatic tissue at different time points in the two groups. Data are presented as mean ± SD. An unpaired two-tailed Student’s *t*-test was applied in GraphPad Software for the MC concentration of the T group at two time points. Abbreviations: DW, dry weight; -, not detected; NT, the non-toxic cyanobacteria group; T, the toxin-producing cyanobacteria group; **** *p* < 0.0001.

Groups	MCs Concentration (μg · g^−1^ DW)			Unpaired *t*-Test	
	0 d	7 d	14 d	R^2^	*p*
NT	-	-	-	-	-
T	-	2.41 ± 0.12	1.43 ± 0.10	0.961	<0.0001 ****

**Table 2:** Results of PERMANOVA, ANOSIM, and MRPP tests between the two groups at each time point. *p*-values less than 0.05 are shown in bold. * *p* < 0.05.

Times	PERMANOVA		ANOSIM		MRPP	
	R^2^	*p*	R	*p*	A	*p*
7 d	0.091	0.784	−0.080	0.698	−0.013	0.802
14 d	0.254	0.035 *	0.496	0.022 *	0.095	0.029 *

**Table 3:** Key topological parameters of the co-occurrence network of gut microbes in each group. Abbreviations: ACC, average clustering coefficient; APL, average path length.

Groups	Nodes	Edges	Average Degree	ACC	Density	Modularity	Diameter	APL
D7NT	29	65	4.483	0.665	0.160	0.535	8	3.345
D7T	47	383	16.298	0.800	0.354	0.305	6	2.156
D14NT	55	280	10.182	0.759	0.189	0.621	8	3.452
D14T	65	579	21.055	0.766	0.390	0.247	4	1.834

## Data Availability

The data presented in the study are deposited in the NCBI repository, accession number PRJNA913611. All other data are presented in the manuscript or Supplementary Materials.

## Supplementary Materials

The following supporting information can be downloaded at: https://www.mdpi.com/article/10.3390/toxins15040252/s1, Table S1: The number of sequencing reads for each sample after quality control; Table S2: Clustering results for each ZOTU; Table S3: Relative abundance of each group of gut microbes at the phylum level; Table S4: Relative abundance of each group of gut microbes at the genus level; Table S5: Median βNTI values and the percentage contribution of deterministic and stochastic processes in each group; Table S6: The product information for BG-11 medium; Table S7: The product information for NMB3# medium.
